# High-Pressure Game Conditions Affect Quiet Eye Depending on the Player’s Expertise: Evidence from the Basketball Three-Point Shot

**DOI:** 10.3390/brainsci12020286

**Published:** 2022-02-18

**Authors:** Francesco Giancamilli, Federica Galli, Andrea Chirico, Dario Fegatelli, Luca Mallia, Tommaso Palombi, Fabio Lucidi

**Affiliations:** 1 Department of Social and Developmental Psychology, Sapienza University of Rome, 00185 Rome, Italy; federica.galli@uniroma1.it (F.G.); andrea.chirico@uniroma1.it (A.C.); dario.fegatelli@gmail.com (D.F.); tommaso.palombi@uniroma1.it (T.P.); fabio.lucidi@uniroma1.it (F.L.); 2 Department of Movement, Human and Health Sciences, Foro Italico University of Rome, 00135 Rome, Italy; luca.mallia@uniroma4.it

**Keywords:** vision, quiet eye, expertise, gaze behavior, basketball, three-point shot, eye tracking, sport, attention, perception–action

## Abstract

Research on attention in sport using eye-tracking methodology has highlighted that the highest levels of expertise and performance are characterized by a specific gaze behavior consisting of a perception–action variable named quiet eye. The present study aimed to understand the role of quiet eye during the three-point shot, especially in game conditions in which even a single point may determine victory or defeat. Twenty-one basketball players (twelve competitive elites and nine semi-elites) with a high-shooting style performed three-point shots in four game scenarios different from each other for the time available (time pressure) and the relevance of the score (performance pressure). The results showed that competitive elites performed a longer quiet eye online duration and a shorter QE preprogramming duration than semi-elites, especially in the highest-pressure condition. On the one hand, these results suggest that quiet eye during three-point shots could fulfill an online control function. On the other hand, the findings stressed the importance of implementing experimental conditions that can resemble as closely as possible actual sport situations. Finally, we suggest that sport professionals interested in administering to athletes a quiet eye training protocol in order to improve three-point shot performance consider the shooting style of the players.

## 1. Introduction

In 1996, Vickers found that elite basketball players lengthened their last eye fixation before the extension of the arm during successful shots in comparison to unsuccessful shots [1]. Vickers named this fixation “quiet eye” (QE), defining it as “the final fixation or tracking gaze that is located on a specific location or object in the task environment within 3° of visual angle (or less) for a minimum of 100 ms” [2]. In a subsequent work in 2001, Harle and Vickers taught university basketball players how to extend this final fixation. The players who received the training protocol improved their free throw shooting accuracy more than those who did not receive it [3]. A few years later, Vine and Wilson proposed the same training protocol to novice basketball players. Their results showed that the prolongation of QE led not only to an increase of successful free throws but permitted players also to resist the adverse effects of anxiety on free throw performance [4]. The results described so far have been replicated throughout the last 25 years in a wide range of sports (e.g., archery, billiards, golf, soccer, hockey, shotgun) and motor tasks (e.g., targeting, interceptive timing, tactical, cf. [5]). Generally, the literature reports that experts showed earlier and longer QE than near or non-experts, as well as successful performances compared to unsuccessful ones [6,7,8,9,10,11]. Moreover, QE training protocols positively affect aiming performance, cf. [3,4,12,13]. Given the relevance of QE in defining the highest levels of expertise and performance [14], various authors have focused on understanding the underlying mechanisms of this fixation. To date, there is no unanimous agreement in the literature on the prevalent one since QE seems to fulfill more than one function related to expertise and performance [15,16,17,18]. However, several findings suggest that QE could represent a measure of attentional control [15,19]. Following the Attentional Control Theory (ACT; [20,21]), an extended QE permits athletes to inhibit all the irrelevant information, allowing athletes to keep focused on their task without being distracted from internal (e.g., negative self-talk or emotions) or external (e.g., the noise of the crowd in the stands) distractions [2,22,23,24]. Accordingly, QE is related to the preservation of the attentional state of the athletes [23,25,26]. Following the review of Gonzalez and colleagues, an extended QE allows athletes to extract useful environmental information for the task [15]. This information is necessary to plan the movement parameters (i.e., a preprogramming function) and potentially adjust the action taking place (i.e., an online control function; cf. [19,27]. The type of task the athlete is performing seems to influence the gaze behavior e.g., [26,28,29,30,31] and, accordingly, the specific function that QE could fulfill in that task. Several authors have proposed that it is possible to comprehend and analyze QE functions referring to QE timing (when QE begins and ends with respect to the critical movement, cf. [19,27,32,33]. Accordingly, a preprogramming function is related to QE occurring before the critical movement. An online function is associated with QE that occurs during the ongoing action [27,32].

An important example is basketball, a sport that has always been of particular interest in QE literature. Indeed, the type of shot seems to influence the timing of QE [34]. At present, the two most studied basketball shots are the set and the jump shots, cf. [35]. From a kinematic point of view, the difference between these two shots relies on the moment the player shoots the ball. Indeed, in the set shot, the players throw the ball with their feet on the ground. In the jump shot, athletes released the ball during jumping [36]. Studies on the gaze behavior on set shots showed that QE begins in the phase immediately preceding the extension of the arms towards the basket, ending just before their full extension [1]. A long duration of QE characterizes the successful throws and the experts [3,4,31,37,38,39,40,41,42,43]. According to several authors, QE in set shots, given its early timing, plays a relevant role in pre-planning the movement parameters before the critical phase of the movement [15,44].

Jump shots are characterized by a similar timing to set shots for what concerns QE onset. However, unlike set shots, QE could extend throughout and beyond the extension of the arms, especially for high-style shooters (i.e., players who extend their arms when the ball is above the head) rather than low-style shooters (i.e., players who extend their arms when the ball is in front of the face). Indeed, the high-style shot permits players to look at the basket also during the final moments of the shot [29,34,45,46,47,48,49]. Accordingly, the QE characteristics of the jump shots of high-style shooters could represent the acquisition of visual information to control the ongoing action [29,34,45,46,47,48,49,50].

The jump shot is considered a relevant skill in the basketball game, given the advantages of overcoming the opponent’s defense and throwing the ball from various distances [51]. In this regard, it is interesting to note that almost all the research on QE has focused on jump shots performed inside the three-point line (i.e., field goals). The only exception is the work of Vickers and colleagues, who found that elite basketball players with a low-style shooting improved their accuracy during the three-point shot in the case of a long QE duration (QED), with a limited vision of the hoop during the last phases of the action [52].

Despite the significance of the three-point shot in basketball games, it is curious to note that only the study of Vickers and colleagues evaluated QE characteristics of elite low-shooting style players engaged in this task, cf. [52]. In addition to being the shot that provides the most points during a match, the three-point shots play a critical role in establishing the outcome of a game, especially in the fourth game quarter with narrow score differentials [53]. In such a situation, players are subjected to high levels of time and performance pressure. Indeed, the fourth game quarter determines the last opportunity to make throws, in which even a single additional point might determine the outcome of the entire game. The literature on QE has assessed the role of time pressure and performance pressure on QE characteristics, e.g., [41,54], showing that time pressure shortened the QED, negatively influencing performance [54], and that performance pressure could have positive or negative effects on QE characteristics and performance according to the match between task demands and the ability to cope of the players (cf., the biopsychosocial model (BPSM) of challenge and threat; [55,56,57,58]). More specifically, when task demands largely exceed the ability to cope of the players, athletes experience a “threatening state”. In line with ACT and QE’s role on attentional control, in such a state, athletes reduce QED, increasing the proneness to distractions, through an impairment on the goal-directed attentional control, with potential repercussions on performance effectiveness [21,59]. While time and performance pressure are factors that can characterize the shots that occur during the last quarter of a closed-score game, as far as we know, only one previous QE study evaluated the effect of the interaction between time and performance pressure on QE characteristics on set shots (in detail, on the free throws, cf. [60]). The findings showed that the interaction effect of time and performance pressure led players to experience a “threatening state,” with impairment on attentional control, as indicated by the reduction of QED and the delay of QE onset. It is interesting to note that several findings suggest a change in the gaze behavior when task demands are similar to those that athletes can experience during actual game situations [29,52,61], but to our knowledge, no previous QE research on three-point jump shots assessed the stress evaluation process and its effect on attentional control. Indeed, only Vickers and colleagues, and Steciuk and Zwierko [52,62] investigated QE during this type of shot. Still, none of these authors assessed any variables that could relate to the stress evaluation process.

Summarizing all the above, experts and high sports performances are generally characterized by a high level of attentional control [59]. The QE is relevant for the attentional control, permitting the extraction of environmental information across the sport action [15]. Such a process can occur to plan the movement parameters, fulfill a preprogramming function, or monitor the ongoing action, in the case of an online control function [19,29,33,63]. Evidence from the literature suggested that the type of task could determine the specific function fulfilled by QE [26,28,29,30,31]. In particular, QE seems to fulfill an online control function in the motor actions in which the movement permits the observation of the ongoing action (cf. [45]). Several authors suggested that the basketball jump shots relied on this QE function (e.g., [47]). Given the shortage of QE studies on three-point shots, a relevant jump shot in basketball, we aimed to assess the effect of high-pressure conditions, such as those that could occur in the last quarter of a basketball game with narrow score differentials [53]. Such situations are characterized by high levels of time and performance pressure. Accordingly, building on QE literature which assessed the effect of high-pressure conditions (e.g., [19,41,54,64]), and those that suggested a change in QE characteristics when task demands are similar to actual game situations [29,52,61] we explored the effect of time and performance pressure on QE characteristics (cf. [60]) during three-point shots performed using a high-shooting style technique.

Consequently, we assessed QE characteristics (duration and timing), performance accuracy, and the match between task demands and the ability to cope of players with different expertise levels, who conducted three-point shots in counterbalanced conditions, different from each other according to time pressure and performance pressure manipulations. The primary purpose was to investigate the impact of the manipulations we implemented (i.e., time and performance pressure) on QE characteristics, considering all their possible combinations (Table 1). We assumed that time and performance pressure would lead players to experience a threatening state (i.e., the task demands exceed the ability to cope with them, cf. [21]), impairing QE characteristics and shot accuracy [41,54,60]. Given the literature on the gaze behavior of high-style shooters (e.g., [48]), we expected that the impairment on QE characteristics could especially affect QE late components (i.e., QE offset and QE online duration, cf. [19]). In the second place, we aimed to explore the effect of time and performance pressure according to the expertise level of the players. Accordingly, we expected that the time and performance pressure would have a greater impact on the minor expert players’ accuracy and QE. In contrast, the most expert athletes would exhibit higher accuracy and superior attentional control, regardless of the manipulations (cf. [59]).

## 2. Materials and Methods

### 2.1. Participants

We used convenience sampling for the current study. Indeed, we contacted a local basketball team that had already collaborated with us in our previous work [60]. We asked coaches about athletes’ reachability, and we successfully recruited 21 male basketball players. To note, all the players were the same who participated in the data collection of our previous work already mentioned (cf. [60]), except for the participant “P20”. However, it is essential to underline that the data collection reported in the present work and the task requested of the athletes were different from those of Giancamilli and colleagues [60].

The expertise of each player was categorized according to the equation and classification system of [65], which used: (A) The athlete’s highest standard of performance, (B) the success at the athlete’s highest level, (C) the experience at the athlete’s highest level, (D) the competitiveness of sport in the athlete’s highest level and (E) the global competitiveness of sport. Accordingly, our sample is composed of nine semi-elite and twelve competitive-elite players. All players used the high-style shooting technique and self-reported normal vision.

From a descriptive point-of-view, the so-called “semi-elite” participants played from regional divisions to the 4th National Italian Amateur division (from Regional divisions to Italian Serie C Gold). The mean age of the semi-elite group was 13.78 years (SD = 1.56). They had an average of 4.06 years of playing experience (SD = 2.19), and they trained an average of 4.22 days per week (SD = 1.56).

Concerning the group of so-called “competitive-elite” participants, it is composed of athletes who played in the 1st to 3rd National Italian Basketball division (Italian series B, A2, and A). The mean age of the competitive-elite group was 16.92 years (SD = 1.78). They had an average of 9.33 years of playing experience (SD = 3.52), and they trained an average of 6.25 days per week (SD = 1.23). The Ethical Committee of the Department of Psychology of Development and Socialization Processes (“Sapienza” University of Rome) approved the present study before participant recruitment. We collected written informed consent from all participants. In the case of minors, we collected informed consent from parents or legal guardians.

### 2.2. Equipment

All the shots were recorded using a digital high-definition camera (HDR-PJ410, Sony, Japan) located orthogonal to the shooting trajectory to determine each phase of the shooting movement. The gaze behavior was recorded using a SensoMotoric Instruments (SMI) light head-mounted mobile binocular eye tracker with automatic parallax compensation. The specific model used for the present study was the Eye Tracking Glasses 2 (SMI ETG 2, SensoMotoric Instruments GmbH, Teltow, Germany) with a sampling rate of 60 Hz. The SMI ETG 2 weighs approximately 68 grams. It is composed of goggles with two infrared cameras to record eye movement, with a gaze tracking accuracy of 0.5° of visual angle. A third high-definition camera is in the central part of the goggles, above the nose pad, to record the visual scene. The SMI ETG 2 implemented a proprietary algorithm (i.e., “SMI Event Detection algorithm”) based on a velocity-based algorithm [66], (pp. 316–319) to detect the initiation and the end of each fixation. For the present data collection, the resolution of the visual scene camera was set at 960 × 720p @ 30fps. An external recording unit (Galaxy Note 4 smartphone, Samsung) was placed in a small waist bag attached to the lower backs of participants and linked via USB to the SMI ETG 2 to improve the players’ comfort. The external recording unit was remotely controlled through a laptop (Lenovo Thinkpad X230; Lenovo, Hong Kong, China) used for calibrating and monitoring the participants’ gaze behavior. The calibration and the real-time monitoring of the gaze behavior were performed using the iViewETG software (iViewETG SMI; version 2.7.0). We calibrated the eye-tracker by asking players to look on a specific corner on the basketball backboard. The calibration was mandatory since the SMI ETG 2 requested a reference point before recording gaze movements. After the data collection, we extracted the gaze behavior data from the recordings using the SMI BeGaze software (version 3.7.60).

### 2.3. Measures

#### 2.3.1. Shot Accuracy

The performance was scored using a 4-point scale used in previous QE basketball studies (e.g., [31]). A hit without rim contact is coded as 4 points, while with board or rim contact it is 3 points; a shot that fails, missing the board or the rim is 2 points, and an airball is 1 point. Accordingly, all the shots coded as 4 and 3 points are labeled as “successful shots,” and all the shots with a score of 2 and 1 points as “unsuccessful shots”. Moreover, we measured the shooting percentage (i.e., ratio of successful shots to the number of total shots performed).

#### 2.3.2. Action Time

We used the video file from the camcorder to code each shot in three phases, and according to QE literature, we referred to the arms’ movements (cf. [29,49,52]). The onset of each shot occurred when the player held the ball with both hands. Accordingly, we coded the “jump phase” starting with the first video frame in which the player made the first upward movement of the arms to raise the ball in the direction of the basket. The “flexion phase” was immediately after the “jump phase,” beginning with the first video frame in which the player flexed the elbow, until its maximum flexion, with the ball raised above the head. The “extension phase” had its onset immediately after the “flexion phase,” and it started as soon as the player began to increase the angle between the upper and the lower arm, ending with the complete extension of the arm, with the ball leaving the fingertips. The action time was computed in milliseconds as the difference between the jump phase’s onset and the extension phase’s end.

#### 2.3.3. Demand and Resource Evaluation Score (DRES) and Trait Anxiety (STAI-Y2)

We measured the perceived task demand and perceived resource to perform the task in a modality in line with previous studies [56,67]. The perceived task demands were assessed by asking, “How difficult do you consider the next condition?”. The rating scale was a 10-point Likert scale (1 = not at all; 10 = extremely). The item to assess the perceived resource to perform the task was drawn from the Mental Readiness Form-Likert (MRF-L; [68]). In detail, we employed the MRF-L self-confidence item, which is composed of a bipolar 11-point Likert scale (confident/not confident) in which participants report how they feel “right now”. Before computing the DRES, we transformed the 11-point Likert scale to a 10-point one, and we reversed the new item (i.e., from “confident/not confident” to “not confident/confident”). The DRES was calculated by subtracting perceived task demands from perceived resources, and the results were normalized in Z scores to make the findings easier to understand. A DRES equal to or close to zero should reflect a challenge state, indicating perceived resources that equal or are very near to perceived task demands. It is worth noting that a large overflow of perceived resources compared to perceived demands should signify disengagement from the activity. On the other hand, a negative score should indicate a threat state (i.e., the perceived task demands exceed the perceived resources). While there is an absence of psychometric testing with the DRES, Brimmell and colleagues [56] observed that this measure had been used in QE research [56,67] and is connected to performance across many tasks [69].

Given that people with higher trait anxiety could tend to perceive situations as more threatening than people with low trait anxiety (e.g., [70,71,72]), we also used the STAI-Y2 form of the State Trait Anxiety Inventory-Y [73,74,75]. The STAI-Y2 is a self-administered questionnaire with 20 questions that measure the person’s overall anxiety levels. Higher scores indicate higher levels of trait anxiety. The STAI-Y2 score was used in the analysis to control for the effect of trait anxiety on the DRES score.

#### 2.3.4. QE Onset

The initiation of QE is called “QE onset,” and it occurs before performing the critical movement. The extension of the arm before the release of the ball has been defined as the critical movement in basketball throws (i.e., the onset of the extension phase [3,4,40,41,42]). Therefore, we calculated QE onset as the interval in milliseconds between the onset of the extension phase and QE initiation. A negative value indicates that QE began before the critical movement.

#### 2.3.5. QE Duration

To be considered a QE, a fixation had to last at least 100 milliseconds, within 3° of visual angle, and be the last fixation directed at the rim, the backboard, or the net [29,42] immediately before the critical movement (i.e., the onset of the extension phase). For the present study, QE duration was calculated as the difference between the end of QE and the initiation of QE.

#### 2.3.6. QE Offset

The end of QE is called “QE offset”. It occurs when the gaze deviates off the location for a minimum of 100 ms [14]. We calculated this variable as the interval, in milliseconds, between the onset of the extension phase and the end of QE. A positive value represents that QE ends after the critical movement (i.e., the onset of the extension phase).

#### 2.3.7. QE Preprogramming and Online Duration

Similar to [19,32], the contribution of QE for preprogramming or online purposes has investigated computing two specific QE components. The QE preprogramming duration is defined as the interval, in milliseconds, starting at QE onset and ending at the initiation of the action (i.e., the onset of the jump phase). The QE online duration is defined as the interval, in milliseconds, starting at the initiation of the action and ending at QE offset.

### 2.4. Task and Protocol

The protocol adopted was the same as employed in our previous work [60]. We collected the data in a basketball court compliant with the Italian Basketball Federation (FIP) normative. Each participant was informed through a written informed consent regarding the present study in terms of the general aim and the procedure adopted before taking part in the data collection. The STAI-Y2 was then administered. After completing the STAI-Y2, we showed the equipment used, permitting participants to ask any questions. Once this briefing phase was completed, we initiated the warm-up phase, which consisted of 10 min during which each participant conducted his usual warm-up routines and basketball three-point shots. At the end of the 10 min, the researchers helped the athletes put on the SMI ETG 2, requesting each participant to perform not less than ten three-point shots. In doing so, we permitted athletes to get used to the instrumentation, and we verified the proper functioning of the SMI ETG 2 before the collection of gaze behavior data. The participants could continue to conduct shots at their leisure as soon as they feel confident with the equipment. We began collecting data when the participants had become comfortable with the method and equipment. The participants’ task consisted in performing ten three-point shots in each of the four conditions (without time pressure and performance pressure: NOTP/NOPP; with performance pressure and without time pressure: NOTP/PP; with time pressure and without performance pressure: TP/NOPP; with time pressure and performance pressure: TP/PP), for a total of 40 shots per participant. In detail, we employed a partial counterbalanced design, so each participant performed the four conditions with a different order (e.g., participant “P1” performed the conditions in the following order: (1) NOTP/NOPP, (2) NOTP/PP, (3) TP/NOPP, (4) TP/PP; participant “P2” performed the conditions in the following order: (1) NOTP/PP, (2) TP/NOPP, (3) TP/PP, (4) NOTP/NOPP; and so on for the other participants).

Before each condition, one of the scholars had the role of giving participants the condition instructions (Table 1) and administering the measures required to compute the DRES. The same scholar made the players believe that he scored only the shots during the conditions with performance pressure (NOTP/PP and TP/PP ones) to build the public ranking. After providing the condition’s related instructions and administering the questionnaire, we placed a bucket containing not less than ten basketball balls close to the participants. Another scholar involved in the data collection had the role of remaining near the participant to pass the balls. Note that the researcher did not enter the visual field of the athlete engaged in the condition, but he was sufficiently near to grab the ball from the bucket and pass it to the player. Such task of the scholar was particularly relevant during conditions with time pressure (TP/NOPP and TP/PP ones). In these conditions, the researcher was instructed to pass the ball using a rhythmic and fast pace to the participant involved in the conditions with time pressure. All shots were taken from behind the three-point shot line (distance from the center of the basket = 6.75 m), in the position straight in front of the basket. Another researcher, different from the other two, calibrated the SMI ETG 2 and continuously verified the calibration quality in real time during data collection until the condition’s completion to guarantee its stability. A fourth scholar had the role of starting the camcorder and checking the proper functioning of the instrument. We gave participants a 5-min break between conditions to avoid fatigue issues. The total procedure took approximately 60 min.

### 2.5. Data Analysis

The video data produced by the SMI-ETG 2 and the digital camera were manually synced frame-by-frame by identifying a specific event observable in both the video files (e.g., the ball touching the ground or the rim, cf. [29]) using the SMI BeGaze software for the gaze behavior video and the VideoPad software (NCH Software, version 10.36) for the participant’s movement video. The video file synchronization and the coding of each action phase were performed by two coders working together. Each stage of these procedures ended only after a unanimous agreement between the coders. The gaze behavior data were then extracted from the selected areas of interest (AOIs), which were the rim, the backboard, and the net (cf. [29,42]) using the “AOI editor” and “Export Metrics” functions provided by BeGaze. The number of shots coded for the analysis was 840, performed by twelve competitive elites and nine semi-elites (Table 2).

The dependent measures of the current study were analyzed employing several mixed-models ANOVA with fixed and random effects. We considered as fixed effects the expertise participants (competitive elite; semi-elite), the time pressure (NOTP = without time pressure; TP = with time pressure), the performance pressure (NOPP = without performance pressure; PP = with performance pressure), and the throw outcome (hit; miss); the random effects of the models were participants (considered as random intercept; n = 21). Analyzing the DRES, we employed the STAI-Y2 score as a covariate, also removing the fixed effect “throw outcome” given that DRES was a condition-related variable. We removed the fixed effect “throw outcome” for what concerns the analysis of shot accuracy in terms of points. Instead, for what concerns the analysis on shot accuracy in terms of shooting percentage, we employed a two-way mixed ANOVA with a between-subject factor “expertise” (two levels: Competitive elite; semi-elite) and a within-subject factor “condition” (four levels: NOTP/NOPP; NOTP/PP; TP/NOPP; TP/PP).

In the presence of significant interaction effects, post hoc pairwise comparisons were employed using the Bonferroni correction to determine interaction effects. Data were analyzed using IBM SPSS version 27 [76]. The software automatically employed the Satterthwaite approximation to calculate the degrees of freedom. The significance level was set at α = 0.05; meanwhile, α levels between 0.05 and 0.10 are considered marginally significant. At last, the effect size of each ANOVA effect was calculated using partial eta squared (η^2^*_p_*) through the calculator provided by [77]. The effect size was interpreted according to Cohen’s criteria (cf. [78,79]), with 0.0099 considered a low effect, 0.0588 a medium effect, and 0.1379 a large effect.

## 3. Results

Due to the high number of analyzed effects, the non-statistically significant effects and the statistics not reported in the “Results” section are shown in Appendix A (Table A1, Table A2, Table A3, Table A4, Table A5, Table A6, Table A7 and Table A8). Moreover, we reported the post hoc pairwise comparisons only for the highest order significant interaction effects. However, we reported the complete descriptive statistics of mixed-models ANOVA with fixed and random effects for each dependent variable in Appendix B (Table A9, Table A10, Table A11, Table A12, Table A13 and Table A14).

### 3.1. Shot Accuracy

#### 3.1.1. Points

**Figure 1:**
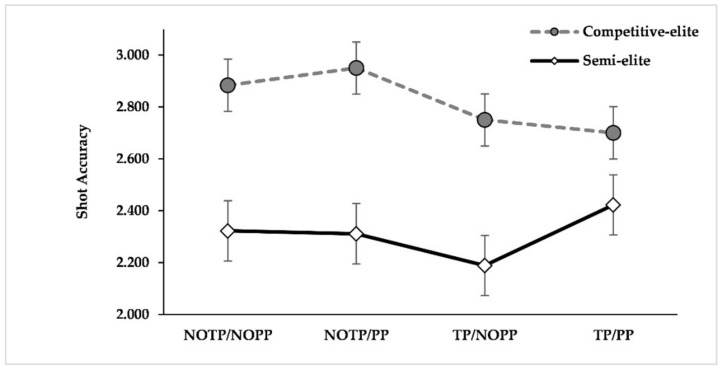
Average values of shot accuracy (in terms of points) for each condition according to expertise levels. The bars represent the standard errors. NOTP = No Time Pressure; NOPP = No Performance Pressure; TP *=* Time Pressure; PP = Performance Pressure.

ANOVA results showed a significant difference for expertise (F_(1, 21)_ = 22.562, *p* < 0.001, η^2^*_p_ =* 0.518). Regardless of the manipulations implemented, competitive elites had a higher shot accuracy than semi-elites (competitive elites: M = 2.821, SE = 0.070; semi-elites: M = 2.311, SE = 0.081). The results did not show other significant effects. The average values of shot accuracy of each condition according to expertise levels are shown in Figure 1.

#### 3.1.2. Shooting Percentage

ANOVA results showed a significant difference for expertise (F_(1, 19)_ = 20.282, *p* < 0.001, η^2^*_p_ =* 0.516). Regardless of the condition, competitive elites had a higher shooting percentage than semi-elites (competitive elites: M = 45.6%, SE = 3%; semi-elites: M = 25.3%, SE = 3.4%). The findings did not show significant effects in terms of the main effect of the condition (F_(3, 57)_ = 1.350, *p* = 0.267, η^2^*_p_ =* 0.066) and in terms of the interaction effect of the expertise × condition (F_(3, 57)_ = 1.776, *p* = 0.162, η^2^*_p_ =* 0.085). The average values of shooting percentage of participants for each condition and according to expertise levels are shown in Table 3.

### 3.2. Action Time

Findings exhibited a significant effect for performance pressure (F_(1, 819_._070)_ = 9.426, *p* < 0.01, η^2^*_p_ =* 0.011) and significant interaction effects about expertise × time pressure (F_(1, 819_._123)_ = 11.728, *p* < 0.01, η^2^*_p_ =* 0.014), expertise × performance pressure (F_(1, 819_._070)_ = 8.571, *p* < 0.01, η^2^*_p_ =* 0.010), time pressure × performance pressure (F_(1, 819_._275)_ = 7.387, *p* < 0.01, η^2^*_p_ =* 0.009), expertise × time pressure × performance pressure (F_(1, 819_._275)_ = 13.651, *p* < 0.001, η^2^*_p_ =* 0.016), expertise × performance pressure × throw outcome (F_(1, 819_._601)_ = 4.240, *p* < 0.05, η^2^*_p_ =* 0.015). Pairwise comparisons of expertise × time pressure × performance pressure interaction exhibited that time pressure shortened the action time of semi-elites when performance pressure occurred (*p* < 0.05, NOTP/PP: M = 436.566 ms, SE = 36.374 ms; TP/PP: M = 395.683 ms, SE = 36.027 ms) and of competitive elites during condition s without performance pressure (*p* < 0.05, NOTP/NOPP: M = 480.661 ms, SE = 30.915 ms; TP/NOPP: M = 450.270 ms, SE = 30.961 ms). Instead, time pressure extended the action time of competitive elites during condition s with performance pressure (*p* < 0.001, NOTP/PP: M = 477.433 ms, SE = 30.921 ms; TP/PP: M = 543.160 ms, SE = 30.992 ms). About the TP/PP condition, the competitive elites performed a longer action time than semi-elites in this condition (*p* < 0.01, competitive elites: M = 543.160 ms, SE = 30.992 ms; semi-elites: M = 395.683 ms, SE = 36.027 ms). Moreover, performance pressure extended the action time of competitive elites during conditions with time pressure (*p* < 0.001, TP/NOPP: M = 450.270 ms, SE = 30.961 ms; TP/PP: 543.160 ms, SE = 30.992 ms). Pairwise comparisons of expertise × performance pressure × throw outcome interaction showed that performance pressure significantly increased the action time of competitive elites, during hits (*p* < 0.001, NOPP: M = 455.237 ms, SE = 31.084 ms; PP: M = 519.540 ms, SE = 31.102 ms) and misses (*p* < 0.05, NOPP: M = 475.694 ms, SE = 30.831 ms; PP: M = 501.053 ms, SE = 30.859 ms). Finally, competitive elites performed a significant longer action time than semi-elites in the hits during performance pressure (*p* < 0.05, competitive elites: M = 519.540 ms, SE = 31.102 ms; semi-elites: M = 408.304 ms, SE = 37.207 ms).

### 3.3. Demand and Resource Evaluation Score (DRES)

**Figure 2:**
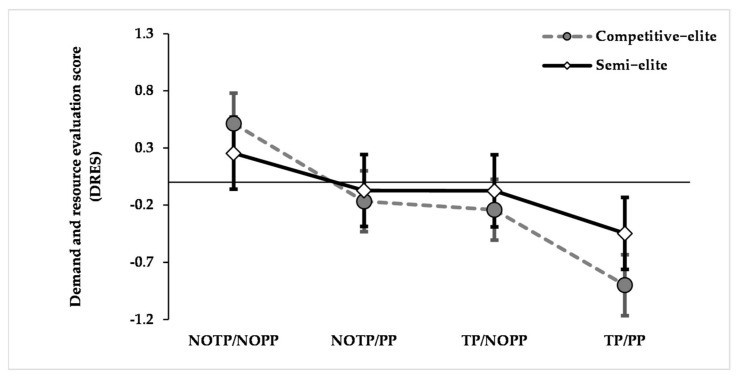
Average DRES for each condition according to expertise levels. The bars represent the standard errors. Note. NOTP = No Time Pressure; NOPP = No Performance Pressure; TP = Time Pressure; PP = Performance Pressure.

Results showed a significant effect for time pressure (F_(1, 819)_ = 334.860, *p* < 0.001, η^2^*_p_ =* 0.290), performance pressure (F_(1, 819)_ = 289.369, *p* < 0.001, η^2^*_p_ =* 0.261), and significant interaction effects about expertise × time pressure (F_(1, 819)_ = 42.794, *p* < 0.001, η^2^*_p_ =* 0.050), and expertise × performance pressure (F_(1, 819)_ = 28.659, *p* < 0.001, η^2^*_p_ =* 0.034). Pairwise comparisons of expertise × time pressure interaction exhibited that time pressure decreased the DRES of the competitive elites (*p* < 0.001, NOTP: M = 0.174, SE = 0.245; TP: M = −0.570, SE = 0.245) and of the semi-elites (*p* < 0.001, NOTP: M = 0.091, SE = 0.290; TP: M = −0.261, SE = 0.290). Also, pairwise comparisons of expertise × performance pressure interaction showed that performance pressure lowered the DRES of the competitive elites (*p* < 0.001, NOPP: M = 0.136, SE = 0.245; PP: M = −0.533, SE = 0.245) and of the semi-elites (*p* < 0.001, NOPP: M = 0.090, SE = 0.290; PP: M = −0.259, SE = 0.290). Moreover, regardless of the expertise, participants showed significant highest DRES in the NOTP/NOPP condition, and the lowest DRES in the TP/PP condition (all *p* < 0.001). No significant differences are found comparing the DRES of the NOTP/PP condition to the TP/NOPP one (all *p* > 0.05) or comparing the DRES of competitive elites and semi-elites for each condition (all *p* > 0.05; Figure 2).

### 3.4. QE Onset

**Figure 3:**
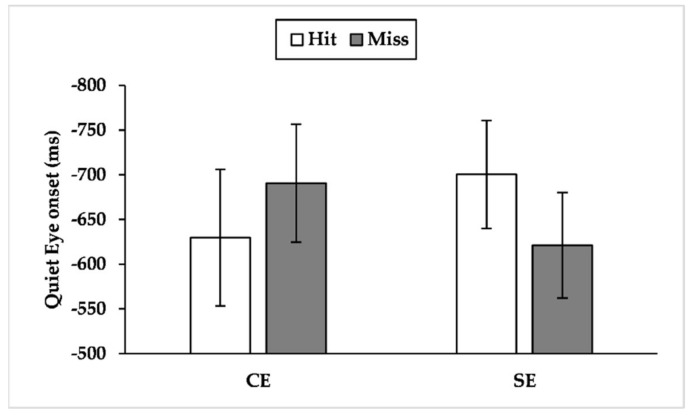
Mean quiet eye onset during hits and misses across expertise levels. Quiet eye onset is reported in milliseconds. A negative value represents a quiet eye onset before the critical movement (i.e., longer bar—earlier quiet eye onset); CE = competitive elite; SE = semi-elite.

**Figure 4:**
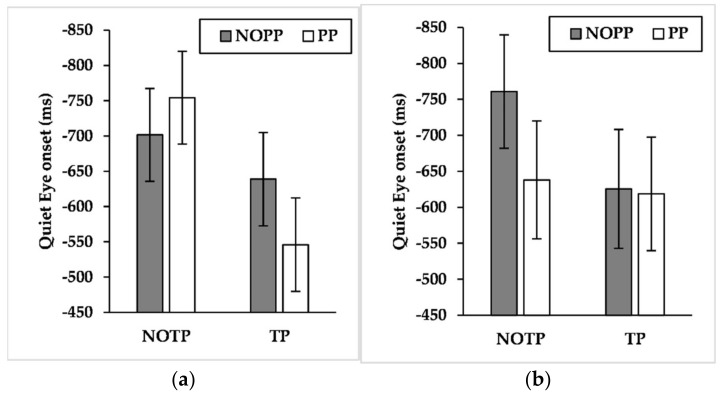
Mean quiet eye onset across time and performance pressure levels by (**a**) competitive-elite and (**b**) semi-elite players. A negative value represents a quiet eye onset before the critical movement (i.e., longer bar—earlier quiet eye onset). NOTP = No Time Pressure; NOPP = No Performance Pressure; TP *=* Time Pressure; PP = Performance Pressure.

Findings showed a significant effect for time pressure (F_(1, 819_._662)_ = 10.088, *p* < 0.01, η^2^*_p_ =* 0.012), a significant interaction effect about expertise × throw outcome (F_(1, 827_._054)_ = 4.202, *p* < 0.05, η^2^*_p_ =* 0.005), and a marginally significant interaction effect about expertise × time pressure × performance pressure (F_(1, 820_._517)_ = 3.797, *p* = 0.052, η^2^*_p_ =* 0.005). Pairwise comparisons of expertise × throw outcome did not exhibit significant differences of QE onset between competitive elites and semi-elites across throw outcome levels, nor between hits and misses across expertise levels. In a purely descriptive way, Figure 3 shows that competitive elites had a later QE onset in their hits than their misses (*p* = 0.141, misses: M = −690.527 ms, SE = 58.973 ms; hits: M = −629.764 ms, SE = 60.290 ms), whereas semi-elites exhibited the opposite pattern (*p* = 0.146, misses: M = −621.234 ms, SE = 65.938 ms; hits: M = −700.305 ms, SE = 76.346 ms). Pairwise comparisons of expertise × time pressure × performance pressure (Figure 4) exhibited that the time pressure delayed QE onset of competitive elites during performance pressure conditions (*p* < 0.001, NOTP/PP: M = −754.327 ms, SE = 65.740 ms; TP/PP: M = −545.856 ms, SE = 66.405 ms).

### 3.5. QE Duration

**Figure 5:**
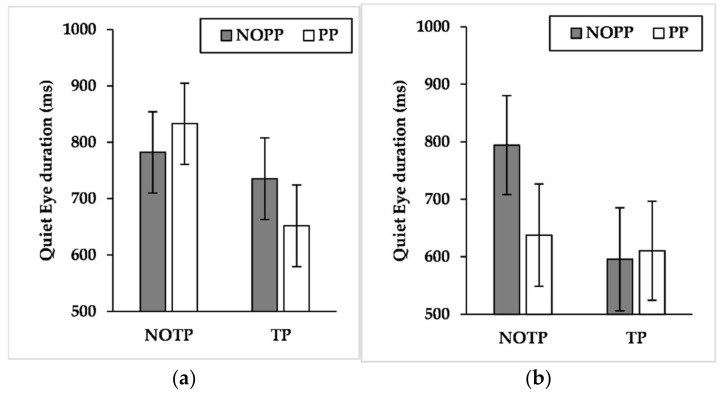
Mean quiet eye duration across time and performance pressure levels by (**a**) competitive-elite and (**b**) semi-elite players. NOTP = No Time Pressure; NOPP = No Performance Pressure; TP *=* Time Pressure; PP = Performance Pressure.

Results showed a significant effect for time pressure (F_(1, 819_._557)_ = 10.969, *p* < 0.01, η^2^*_p_ =* 0.013) and a significant interaction effect concerning expertise × time pressure × performance pressure (F_(1, 820_._268)_ = 4.969, *p* < 0.05, η^2^*_p_ =* 0.006). Pairwise comparisons of expertise × time pressure × performance pressure interaction (Figure 5) exhibited that performance pressure shortened QED of semi-elites during conditions without time pressure (*p* < 0.05, NOTP/NOPP: M = 794.233 ms, SE = 85.994 ms; NOTP/PP: M = 637.707 ms, SE = 89.008 ms). Time pressure reduced QED of semi-elites during conditions without performance pressure (*p* < 0.05, NOTP/NOPP: M = 794.233 ms, SE = 85.994 ms; TP/NOPP: M = 595.657 ms, SE = 89.611 ms) and QED of competitive elites during conditions with performance pressure (*p* < 0.01, NOTP/PP: M = 833.010 ms, SE = 72.003 ms; TP/PP: M = 651.773 ms, SE = 72.637 ms).

### 3.6. QE Offset

**Figure 6:**
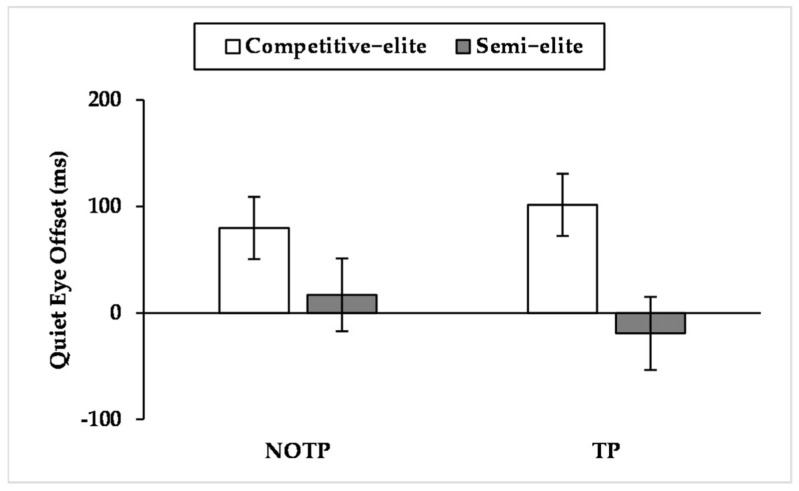
Mean quiet eye offset by expertise levels across time pressure levels. The quiet eye offset is reported in milliseconds. A negative value represents a quiet eye offset that ended before the critical movement. NOTP = No Time Pressure; TP = Time Pressure.

Findings show a significant effect for expertise (F_(1, 21_._444)_ = 4.405, *p* < 0.05, η^2^*_p_ =* 0.170) and a significant interaction effect concerning expertise × time pressure (F_(1, 819_._277)_ = 7.041, *p* < 0.01, η^2^*_p_ =* 0.009). Pairwise comparisons of expertise × time pressure interaction (Figure 6) exhibited that competitive elites performed a later QE offset than semi-elites when time pressure occurred (*p* < 0.05, competitive elites: M = 101.445 ms, SE = 29.241 ms; semi-elites: M = −19.220 ms, SE = 34.288 ms) and that time pressure led semi-elites to anticipate QE offset (*p* < 0.05, NOTP: M = 16.937 ms, SE = 34.254 ms; TP: M = −19.220 ms, SE = 34.288 ms).

### 3.7. QE Preprogramming

**Figure 7:**
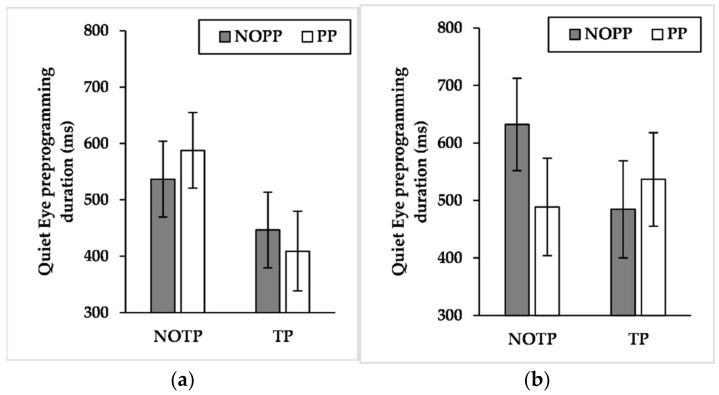
Mean quiet eye preprogramming duration across time and performance pressure levels by (**a**) competitive-elite and (**b**) semi-elite players. NOTP = No Time Pressure; NOPP = No Performance Pressure; TP *=* Time Pressure; PP = Performance Pressure.

Results showed a significant effect for time pressure (F_(1, 629_._886)_ = 6.614, *p* < 0.05, η^2^*_p_ =* 0.01). The findings about interaction effects showed a significant expertise × time pressure × performance pressure (F_(1, 630_._379)_ = 3.910, *p* < 0.05, η^2^*_p_ =* 0.006). Pairwise comparisons of expertise × time pressure × performance pressure interaction (Figure 7) exhibited that the time pressure shortened QE preprogramming duration of competitive elites during conditions with performance pressure (*p* < 0.05, NOTP/PP: M = 587.721 ms, SE = 67.000 ms; TP/PP: M = 408.854 ms, SE = 70.732 ms).

### 3.8. QE Online Duration

**Figure 8:**
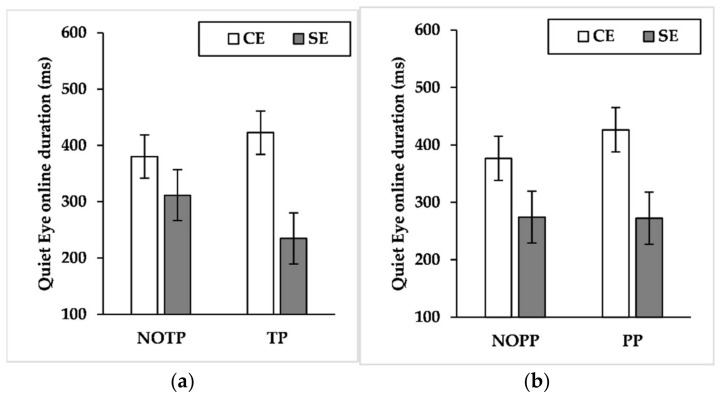
Mean quiet eye online duration by expertise levels across (**a**) time pressure levels and (**b**) performance pressure levels. CE = competitive elite; SE = semi-elite; NOTP = No Time Pressure; TP = Time Pressure; NOPP = No Performance Pressure; PP = Performance Pressure.

**Figure 9:**
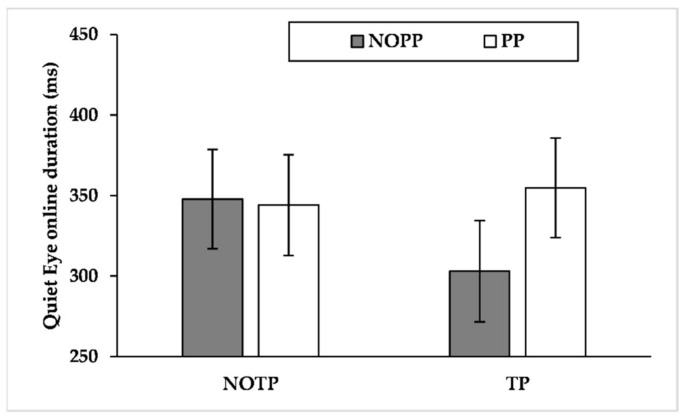
Mean quiet eye online duration by time pressure levels across performance pressure levels. NOTP = No Time Pressure; TP = Time Pressure; NOPP = No Performance Pressure; PP = Performance Pressure.

Findings showed a significant effect for expertise (F_(1, 21_._343)_ = 4.876, *p* < 0.05, η^2^*_p_ =* 0.186) and a marginally significant effect for performance pressure (F_(1, 789_._131)_ = 3.445, *p* = 0.06, η^2^*_p_ =* 0.004). The results about the interaction effects showed significant effects about expertise × time pressure (F_(1, 789_._520)_ = 21.179, *p* < 0.001, η^2^*_p_ =* 0.026), expertise × performance pressure (F_(1, 789_._131)_ = 4.025, *p* < 0.05, η^2^*_p_ =* 0.005), and time pressure × performance pressure (F_(1, 789_._217)_ = 4.624, *p* < 0.05, η^2^*_p_ =* 0.006). Results showed also a marginally significant interaction effect about expertise × time pressure × performance pressure (F_(1, 789_._217)_ = 2.783, *p* = 0.09, η^2^*_p_ =* 0.004). Pairwise comparisons of expertise × time pressure interaction (Figure 8a) exhibited that time pressure led semi-elites to shorten QE online duration (*p* < 0.001, NOTP: M = 291.546 ms, SE = 47.418 ms; TP = 216.273 ms, SE = 47.452 ms), whereas competitive elites exhibited the opposite pattern (*p* < 0.05, NOTP: M = 378.569 ms, SE = 40.570 ms; TP = 417.969 ms, SE = 40.637 ms). Moreover, competitive elites had a longer QE online duration than semi-elites during time pressure (*p* < 0.01, semi-elites: M = 216.273 ms, SE = 47.452 ms; competitive elites: M = 417.969 ms, SE = 40.637 ms). Pairwise comparisons of expertise × performance pressure (Figure 8b) interaction showed that performance pressure produced an extension of QE online duration of competitive elites (*p* < 0.01, NOPP: M = 373.341 ms, SE = 40.593 ms; PP = 423.197 ms, SE = 40.613 ms). Furthermore, competitive elites had an extended QE online duration compared to semi-elites during performance pressure (*p* < 0.05, semi-elites: M = 247.438 ms, SE = 47.415 ms; competitive elites: M = 423.197 ms, SE = 40.613 ms). Pairwise comparisons of time pressure × performance pressure interaction (Figure 9) revealed that the TP/PP condition had, regardless of other factors, longer QE online control than the TP/NOPP condition (*p* < 0.01, TP/NOPP: M = 289.464 ms, SE = 32.729 ms; TP/PP: M = 344.779 ms, SE = 32.404 ms). For what concerns the role of time pressure, this factor led to a diminution of QE online duration, but only without performance pressure (*p* < 0.01, NOTP/NOPP: M = 344.258 ms, SE = 32.348 ms; TP/NOPP: 289.464 ms, SE = 32.729 ms). Pairwise comparisons of expertise × time pressure × performance pressure showed that competitive elites had a longer QE online duration than semi-elites both in the TP/NOPP condition (*p* < 0.05, competitive elites: M = 373.156 ms, SE = 40.073 ms; semi-elites: 232.806 ms, SE = 48.389 ms) and in the TP/PP condition (*p* < 0.001, competitive elites: M = 472.428 ms, SE = 40.075 ms; semi-elites: 237.099 ms, SE = 47.219 ms). Moreover, the performance pressure led to a longer QE online duration only for the competitive elites during time pressure (*p* < 0.001, TP/NOPP: M = 373.156 ms, SE = 40.073 ms; TP/PP: 472.428 ms, SE = 40.075 ms). About the role of time pressure, this factor led to a reduction of QE online duration for the semi-elites during conditions without performance pressure (*p* < 0.05, NOTP/NOPP: M = 315.719 ms, SE = 46.951 ms; TP/NOPP: 232.806 ms, SE = 46.389 ms) and with performance pressure (*p* < 0.05, NOTP/PP: M = 307.549 ms, SE = 48.179 ms; TP/PP: 237.099 ms, SE = 47.219 ms). Instead, the competitive elites extended QE online duration, under the effect of time pressure, during conditions with performance pressure (*p* < 0.001, NOTP/PP: M = 380.498 ms, SE = 39.931 ms; TP/PP: 472.428 ms, SE = 40.075 ms), while no differences emerged during conditions without performance pressure (*p* = 0.749).

## 4. Discussion

In the current study we assessed QE of athletes, with different levels of expertise according to Swann, Moran, and Piggott [65]. We manipulated simultaneously the time provided to athletes (i.e., time pressure) and the relevance of the performance (i.e., performance pressure), also considering the perceived task demands and resources (DRES) during a three-point shot task, with evaluation of both early and late QE components.

We expected that the manipulations that we implemented would affect the appraisal of the stressful situation (i.e., DRES). We hypothesized such an effect given that time and performance pressure are two factors that act during high-pressure conditions, like the ones that may arise in the last quarter of a very close score game [53]. Our results showed that time and performance pressure had a large and significant effect on diminishing the DRES, supporting the effectiveness of the experimental manipulations.

Since the Attentional Control Theory (ACT) predicts that high-pressure conditions would impair the goal-directed attentional system, we assumed consequences on QE characteristics by the effect of time and performance pressure. As we expected, the findings showed that time and performance pressure affected QE characteristics, particularly for the semi-elites. Indeed, semi-elites showed a more unstable QE than competitive elites across the conditions. The findings showed that the effect of a single manipulation (time or performance pressure) was sufficient for the semi-elites to shorten QED. In contrast, competitive elites reduced and delayed QE only in the presence of both time and performance pressure (Figure 3, Figure 4 and Figure 5). In other words, the competitive elites showed a superior attentional control compared to semi-elites across the conditions, in line with the ACT [20,59].

**Figure 10:**
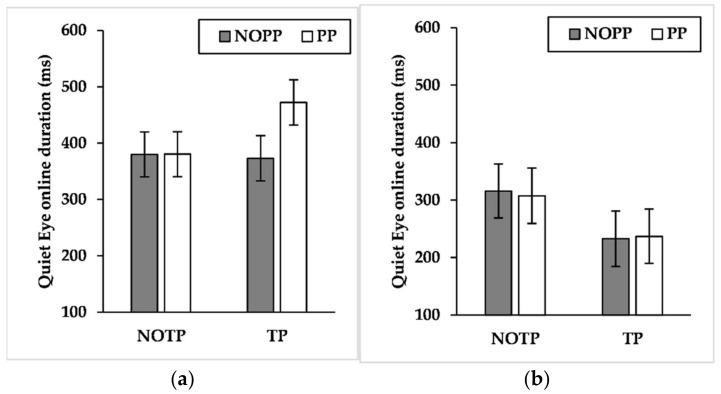
Mean quiet eye online duration across time and performance pressure levels by (**a**) competitive-elite and (**b**) semi-elite players. NOTP = No Time Pressure; NOPP = No Performance Pressure; TP *=* Time Pressure; PP = Performance Pressure.

The large effect sizes of expertise on QE late components (i.e., QE offset and QE online duration) and the small effect size on QED and QE early components (i.e., QE onset and QE preprogramming duration) seems to suggest a relevant role of QE late components on maintaining goal-directed attention during a three-point shot. In this regard, it is interesting to note that competitive elites performed a longer QE online duration than semi-elites, especially when time and performance pressure occurred (Figure 8, Figure 9 and Figure 10). The results about QE offset reported a similar picture, given that during time pressure the competitive elites delayed this QE late component, also performing a later QE offset than semi-elites (Figure 6). Given the results of action time, the extension of QE late components could be aided by an increase of the action time carried out by the competitive elites. Such behavior is comparable to that performed by the sole high-style shooter of [52], who performed an extension of the action time during three-point shots hindered by a defender. Given our results and those of Vickers, Causer, and Vanhooren [52], it is possible to suppose that competitive elites with a high shooting style, engaged in a three-point shot, slow their action speed to watch the basket for a longer time during tough-game conditions. According to our findings, such speculation seems particularly true when successful performances matter, given that competitive elites extended their action time only when time pressure was combined with performance pressure. Instead, the absence of performance pressure led the time pressure to shorten the action time.

Interestingly, while the competitive elites extended QE late time components, especially during harsh game conditions, an opposite trend was observable for QE preprogramming duration. Indeed, while semi-elites maintained a stable QE preprogramming duration across conditions, competitive elites decreased this QE early time component under the influence of time and performance pressure (Figure 7). It is important to note that all the players performed three-point shots using a high shooting style regardless of their expertise. Accordingly, this factor cannot explain the difference in gaze behavior between competitive elites and semi-elite.

Summarizing, the findings of QE characteristics seem to suggest that the late components of this fixation have a relevant role on three-point shots compared to the early components, given that competitive elites had longer QE offset and online duration than semi-elites, particularly in the harshest game conditions. Moreover, a comparison between our sample, composed of high-style shooters, with the one of [52], consisting of low-style shooters, suggests a difference in QE considering the shooting style during three-point shots. Such a statement is in line with the literature on the jump shots and high-style shooters [29,34,45,46,47,48,49,50]. Indeed, this literature suggests that athletes with a high shooting style could monitor the ongoing motor action using late visual information to adjust the action that is taking place [27]. Accordingly, it is possible to speculate that, during three-point shots performed with a high-shooting style, the superior attentional control of the most experienced athletes passes through the late components of QE.

Differently from what we assumed, we did not find a statistically significant detrimental effect of time and performance pressure on shooting accuracy. Indeed, the results showed only a statistically significant higher accuracy of competitive elites than semi-elites, both in terms of points scored and shooting percentage (Figure 1 and Table 3). However, in terms of the shooting percentage, it is possible to note that the conditions in which the manipulations were present showed a deflection of the performance on three-point shots (Table 3), but this deflection was not statistically significant. Given all the above, it is possible to suppose that athletes performed fewer hits than misses in conditions in which at least time or performance pressure were present (cf. Table 2 and Table 3). At the same time, athletes performed the hits on these conditions with a high shot accuracy (e.g., a hit without rim contact). Even if different from what we predicted, the results about shot accuracy are in line with the ACT theoretical framework, which states that anxiety impairs processing efficiency more than performance effectiveness, given that athletes could attempt to compensate by using extra processing resources [59]. Accordingly, it is conceivable to observe a larger impairment of QE characteristics without a consequent significant decline in performance. About the additional processing resources, it is interesting to note that in the condition in which both time and performance pressure were present, competitive elites extended their QE online duration. Such a strategy seems very similar to the one investigated by [64] in some elite biathlon athletes. Their results showed that the athletes who augmented their QED in high-pressure conditions did not experience performance degradation, in contrast to the athletes who did not adopt such strategy [64].

It was also possible to describe an alternative explanation of the not statistically significant detrimental effect of time and performance pressure on shooting accuracy, relying on the findings of the effect sizes. Indeed, the results reported very small effect sizes of time and performance pressure on QE characteristics. Accordingly, it could be possible that the manipulations implemented were not powerful enough to determine a relevant detrimental effect on QE characteristics and consequently on shooting accuracy. Alternatively, it could be possible to speculate that the high level of experience of the participants (competitive elites and semi-elites) could have cushioned the detrimental effect of time and performance pressure on QE characteristics. Indeed, experts can efficiently regulate their affective state (e.g., [80]). Moreover, the relatively low number of shots collected for each condition (i.e., ten shots) may have played a role in determining the not statistically significant effect related to shooting accuracy. Our findings would seem to suggest that future QE literature should also focus on (1) designing experimental settings for getting closer to actual high-pressure sports situations, (2) using instruments to assess not only the challenge or threat states but also the strategies employed to regulate them, (3) collecting as many shots as possible, compatible with the level of fitness of athletes.

In a nutshell, our study investigated the effect of time and performance pressure on three-point shots by the expertise level of the participants. Considering all the above results, the competitive elites are characterized by stable QE characteristics in response to time pressure and performance pressure, and by a longer QE online duration, especially in the harshest game conditions. Such results are in line with the gaze behavior variation when experimental manipulations are similar to actual game demands (cf. [29,52,61]) and QE literature on jump shots and high-shooting style [29,34,45,46,47,48,49,50], suggesting that QE online control function could be particularly relevant in three-point shots performed by athletes with a high-shooting style.

Our work clearly has some limitations. The first refers to the unequal sample size of the groups. We recruited an unequal number of participants according to their expertise level (*n* = 9 semi-elites; *n* = 12 competitive elites), collecting an unequal number of hits and misses (310 hits and 530 misses; Table 2). Accordingly, we employed mixed-models ANOVA with fixed and random effects, given the advantages in terms of unbalanced data sets [81,82,83] and its previous application on QE and gaze behavior research [52,84]. Again, regarding the sample, the two groups analyzed in our study are different in expertise and age. Literature has shown that athletes’ perceptual and cognitive abilities vary according to expertise levels and according to the stage of development (e.g., [85]). Accordingly, the potential role of age in the results of this study should not be excluded.

The second potential limitation of the present study regards the sample size of participants. Drawing from the effect sizes provided by [8] for the between-individuals (i.e., the “expertise” effect: Cohen’s *d* = 1.04, 95% CI [0.71, 1.38]) and the within-individuals (i.e., the “accuracy” effect: Cohen’s *d* = 0.58, 95% CI [0.34, 0.82]) differences in the QE period, we conducted a power analysis using the ‘‘pwr’’ R package [86], employing a significance level equal to 0.05. The results showed that to obtain a power equal to the 80% probability of truly detecting the expertise effect, we should have recruited 12 participants per group. We obtained such a result using the average value of the effect size (*d* =1.04). Instead, using the highest value of the 95% CI (*d* = 1.38), we should have recruited a minimum of seven participants for each expertise level. According to the actual number of participants, we exceeded this latter required sample size. At the same time, we were distant from the necessary sample size according to *d* = 0.71 (*n* = 25 for each group). Concerning the accuracy effect, we calculated that to obtain a power equal to 80%, we should have collected, overall, a minimum of 37 hits and 37 misses (using the average value of the effect size *d* = 0.58). According to the actual number of throws collected, we exceeded the required sample size. Interestingly, the same applies using *d* = 0.34 (the lowest value of the 95% CI). Indeed, the sample size needed according to *d* = 0.34 was equal, overall, to 108 hits and 108 misses. Accordingly, we could state that we reached the minimum sample size of participants to have an 80% power of truly detecting the expertise effect (given a medium-to-large effect: *d* = 0.71). About the accuracy effect, we largely exceeded the 80% power (given a small-to-medium effect: *d* = 0.34).

The third limitation regards the sampling method adopted. Indeed, we implemented a non-probability-based sampling method (convenience sampling). On one side, the present work results should consequently be treated with caution, given that, from a methodological point of view, such a sampling method does not permit generalizing results to the population of interest. On the other hand, it is essential to note that the convenience sampling method is commonly implemented in QE research. Thus, rather than a specific limitation of the present study, implementing a convenience sampling method could be considered an issue globally affecting QE literature. Moreover, our results are in line with those provided by QE literature that investigated this gaze behavior during jump shots and with a high-shooting style. Accordingly, we could affirm that, at least, the sample we collected should not be differently “biased” than other samples available in QE basketball literature.

The fourth and last limitation is about the apparatus. The eye tracker model that we used is very light and comfortable, and it has already been used in QE research on basketball throws [35,87]. However, it should be noted that it is still equipment that athletes do not usually use, so it is plausible to think that it can annoy athletes.

## 5. Conclusions

We aimed to deepen the knowledge on QE fixation in the basketball three-point shot through the current work. We conducted a mixed factorial design research, focusing on the role of expertise and its effect on QE characteristics during high-pressure conditions. The results confirmed previous evidence about QE behavior during jump shots performed with a high shooting style, extending the results of such literature to three-point shots. Moreover, the results are in line with the ACT, confirming the superiority of the attentional control of the highest expertise levels. A core finding of the present work is that the online function of QE seems to have a relevant role in the conditions that resemble those of an actual sport situation. We believe that our work could be helpful to scholars interested in QE and the entourage of basketball athletes, mainly coaches and sports psychologists. The former should care about creating conditions as similar as possible to actual game situations, to bring out ocular behaviors like those that the athletes would perform in real competition, for the benefit of the external validity of their results. The latter should instead focus on implementing a QE training program according to the shooting style of players to overcome the deleterious effect on attentional processes and performance of harsh game conditions.

## Figures and Tables

**Table 1:** Summary of designs of conditions with instructions.

Condition	Time Pressure	Performance Pressure	Condition’s Instructions
NOTP/NOPP	Absent	Absent	“Perform ten three-point shots.”
NOTP/PP	Absent	Present	“Perform ten three-point shots. It is very important to score as many points as you can because your score will be recorded to establish a ranking with your teammates.”
TP/NOPP	Present	Absent	“Perform ten three-point shots as fast as possible.”
TP/PP	Present	Present	“Perform ten three-point shots as fast as possible. It is very important to score as many points as you can because your score will be recorded to establish a ranking with your teammates.”

**Table 2:** Numbers of throws across expertise levels and of the total sample.

Sample	Condition	Throw Outcome		
		Hit	Miss	Total
Competitive-elite	NOTP/NOPP	60	60	120
	NOTP/PP	64	56	120
	TP/NOPP	49	71	120
	TP/PP	46	74	120
	Total	219	261	480
Semi-elite	NOTP/NOPP	26	64	90
	NOTP/PP	20	70	90
	TP/NOPP	19	71	90
	TP/PP	26	64	90
	Total	91	269	360
Total sample	NOTP/NOPP	86	124	210
	NOTP/PP	84	126	210
	TP/NOPP	68	142	210
	TP/PP	72	138	210
	Total	310	530	840

Note. NOTP = No Time Pressure; NOPP = No Performance Pressure; PP = Performance Pressure; TP = Time Pressure.

**Table 3:** Shooting percentage of participants for each condition.

Expertise	Participant	Conditions			
		NOTP/NOPP	NOTP/PP	TP/NOPP	TP/PP
Competitive-elite	P1	60%	50%	70%	50%
	P2	40%	70%	20%	30%
	P3	50%	60%	30%	10%
	P4	40%	30%	40%	40%
	P5	40%	50%	70%	10%
	P6	50%	50%	20%	40%
	P7	70%	70%	70%	80%
	P8	50%	60%	50%	70%
	P9	40%	40%	50%	30%
	P10	50%	30%	30%	30%
	P11	40%	70%	20%	30%
	P12	70%	60%	20%	40%
	Average	50.00%	53.33%	40.83%	38.33%
	SD	11.28%	14.35%	20.65%	20.82%
Semi-elite	P13	10%	10%	20%	10%
	P14	20%	0%	20%	10%
	P15	60%	30%	20%	20%
	P16	20%	10%	10%	50%
	P17	50%	10%	0%	40%
	P18	20%	30%	30%	60%
	P19	30%	40%	30%	20%
	P20	20%	20%	20%	30%
	P21	30%	50%	40%	20%
	Average	28.89%	22.22%	21.11%	28.89%
	SD	16.16%	16.41%	11.67%	17.64%
Total Sample	Average	40.95%	40.00%	32.38%	34.29%
	SD	17.00%	21.68%	19.72%	19.64%

Note. NOTP = No Time Pressure; NOPP = No Performance Pressure; PP = Performance Pressure; TP = Time Pressure; SD = Standard Deviation.

## Data Availability

The data presented in this study are available in: https://osf.io/cnjxp/?view_only=b396782974b14330920bb0fd3d85621a (accessed on 4 January 2022).

## Appendix A

**Table A1:** Statistical outputs of the not statistically significant effects of the mixed-model ANOVA, with fixed and random effects, for the action time.

Results	df, df Error	F	*p*-Value	Partial η²
EXP	1, 21.193	2.487	0.130	0.105
TP	1, 819.123	1.129	0.288	0.001
TO	1, 820.537	0.112	0.738	0.000
EXP × TO	1, 820.537	0.042	0.837	0.000
TP × TO	1, 819.883	0.364	0.547	0.000
PP × TO	1, 819.601	0.278	0.598	0.000
EXP × TP × TO	1, 819.883	0.000	0.996	0.000
TP × PP × TO	1, 820.218	0.062	0.803	0.000
EXP × TP × PP × TO	1, 820.218	0.391	0.532	0.000

Note. EXP = Expertise; TP = Time Pressure; TO = Throw Outcome; PP = Performance Pressure.

**Table A2:** Statistical outputs of the not statistically significant effects of the mixed-model ANOVA, with fixed and random effects, for the shot accuracy.

Results	df, df Error	F	*p*-Value	Partial η²
TP	1, 819	2.552	0.111	0.003
PP	1, 819	0.885	0.347	0.001
EXP × TP	1, 819	2.023	0.155	0.002
EXP × PP	1, 819	0.656	0.418	0.001
TP × PP	1, 819	0.253	0.615	0.000
EXP × TP × PP	1, 819	2.023	0.155	0.002

Note. EXP = Expertise; TP = Time Pressure; PP = Performance Pressure.

**Table A3:** Statistical outputs of the not statistically significant effects of the mixed-model ANOVA, with fixed and random effects, for the demand and resource evaluation score.

Results	df, df Error	F	*p*-Value	Partial η²
EXP	1, 21	0.076	0.786	0.004
TP × PP	1, 819	0.035	0.851	0.000
EXP × TP × PP	1, 819	0.317	0.574	0.000

Note. DRES = Demand and resource evaluation score; EXP = Expertise; TP = Time Pressure; PP = Performance Pressure.

**Table A4:** Statistical outputs of the not statistically significant effects of the mixed-model ANOVA, with fixed and random effects, for the quiet eye onset.

Results	df, df Error	F	*p*-Value	Partial η²
EXP	1, 22.077	0.000	0.994	0.000
PP	1, 819.361	1.605	0.206	0.002
TO	1, 827.054	0.072	0.788	0.000
EXP × TP	1, 819.662	0.753	0.386	0.001
EXP × PP	1, 819.361	0.445	0.505	0.001
TP × PP	1, 820.517	0.048	0.826	0.000
TP × TO	1, 823.806	0.799	0.372	0.001
PP × TO	1, 822.314	1.956	0.162	0.002
EXP × TP × TO	1, 823.806	0.453	0.501	0.001
TP × PP × TO	1, 825.514	0.001	0.977	0.000
EXP × PP × TO	1, 822.314	0.329	0.566	0.000
EXP × TP × PP × TO	1, 825.514	0.172	0.678	0.000

Note. QE = Quiet Eye; EXP = Expertise; PP = Performance Pressure; TP = Time Pressure; TO = Throw Outcome.

**Table A5:** Statistical outputs of the not statistically significant effects of the mixed-model ANOVA, with fixed and random effects, for the quiet eye duration.

Results	df, df Error	F	*p*-Value	Partial η²
EXP	1, 21.899	0.884	0.357	0.039
PP	1, 819.307	1.625	0.203	0.002
TO	1, 825.807	0.003	0.955	0.000
EXP × TP	1, 819.557	0.000	0.987	0.000
EXP × PP	1, 819.307	0.630	0.427	0.001
EXP × TO	1, 825.807	2.688	0.101	0.003
TP × PP	1, 820.268	0.073	0.788	0.000
TP × TO	1, 823.028	0.638	0.425	0.001
PP × TO	1, 821.769	1.327	0.250	0.002
EXP × TP × TO	1, 823.028	0.168	0.682	0.000
TP × PP × TO	1, 824.48	0.104	0.747	0.000
EXP × PP × TO	1, 821.769	0.003	0.953	0.000
EXP × TP × PP × TO	1, 824.48	0.102	0.749	0.000

Note. Quiet Eye Duration; EXP = Expertise; PP = Performance Pressure; TO = Throw Outcome; TP = Time Pressure.

**Table A6:** Statistical outputs of the not statistically significant effects of the mixed-model ANOVA, with fixed and random effects, for the quiet eye offset.

Results	df, df Error	F	*p*-Value	Partial η²
TP	1, 819.277	0.433	0.511	0.001
PP	1, 819.152	0.010	0.919	0.000
TO	1, 822.528	0.339	0.561	0.000
EXP × PP	1, 819.152	0.209	0.648	0.000
EXP × TO	1, 822.528	1.367	0.243	0.002
TP × PP	1, 819.634	2.399	0.122	0.003
TP × TO	1, 821.045	0.086	0.770	0.000
PP × TO	1, 820.394	0.433	0.511	0.001
EXP × TP × PP	1, 819.634	1.029	0.311	0.001
EXP × TP × TO	1, 821.045	0.488	0.485	0.001
TP × PP × TO	1, 821.81	0.686	0.408	0.001
EXP × PP × TO	1, 820.394	2.292	0.130	0.003
EXP × TP × PP × TO	1, 821.81	0.067	0.795	0.000

Note. QE = Quiet Eye; TP = Time Pressure; PP = Performance Pressure; TO = Throw Outcome; EXP = Expertise.

**Table A7:** Statistical outputs of the not statistically significant effects of the mixed-model ANOVA, with fixed and random effects, for the quiet eye preprogramming duration.

Results	df, df Error	F	*p*-Value	Partial η²
EXP	1, 21.317	0.216	0.647	0.010
PP	1, 630.059	0.301	0.584	0.000
TO	1, 635.397	0.083	0.773	0.000
EXP × TP	1, 629.886	1.391	0.239	0.002
EXP × PP	1, 630.059	0.536	0.464	0.001
EXP × TO	1, 635.397	2.391	0.123	0.004
TP × PP	1, 630.379	0.552	0.458	0.001
TP × TO	1, 633.510	0.393	0.531	0.001
PP × TO	1, 630.790	1.085	0.298	0.002
EXP × TP × TO	1, 633.510	0.013	0.908	0.000
TP × PP × TO	1, 635.075	0.060	0.806	0.000
EXP × PP × TO	1, 630.790	0.130	0.719	0.000
EXP × TP × PP × TO	1, 635.075	0.002	0.965	0.000

Note. QE = Quiet Eye; EXP = Expertise; PP = Performance Pressure; TO = Throw Outcome; TP = Time Pressure.

**Table A8:** Statistical outputs of the not statistically significant effects of the mixed-model ANOVA, with fixed and random effects, for the quiet eye online duration.

Results	df, df Error	F	*p*-Value	Partial η²
TP	1, 789.520	1.734	0.188	0.002
TO	1, 791.456	0.136	0.712	0.000
EXP × TO	1, 791.456	0.717	0.397	0.001
TP × TO	1, 790.853	0.092	0.762	0.000
PP × TO	1, 790.285	0.003	0.953	0.000
EXP × TP × TO	1, 790.853	0.900	0.343	0.001
TP × PP × TO	1, 791.066	1.333	0.249	0.002
EXP × PP × TO	1, 790.285	0.161	0.688	0.000
EXP × TP × PP × TO	1, 791.066	0.368	0.544	0.000

Note. QE = Quiet Eye; TP = Time Pressure; TO = Throw Outcome; EXP = Expertise; PP = Performance Pressure.

## Appendix B

**Table A9:** Average action time in milliseconds across throw outcome, expertise, time pressure, and performance pressure.

Throw Outcome	Expertise	Condition			
		NOTP/NOPP	NOTP/PP	TP/NOPP	TP/PP
Hit	CE	471.457 (32.184)	481.058 (32.036)	439.017 (32.789)	558.023 (32.993)
	SE	428.201 (39.261)	427.911 (40.703)	409.33 (40.938)	388.696 (39.28)
Miss	CE	489.865 (32.184)	473.809 (32.371)	461.523 (31.821)	528.297 (31.734)
	SE	428.158 (36.31)	445.221 (36.132)	394.545 (36.098)	402.67 (36.313)

Note. Standard errors are presented inside the round brackets. Throw outcome (Hit; Miss); Expertise (CE = Competitive-elite; SE = Semi-elite); Time pressure (NOTP = No Time Pressure; TP = Time Pressure); Performance pressure (NOPP = No Performance Pressure; PP = Performance Pressure).

**Table A10:** Average quiet eye onset in milliseconds across throw outcome, expertise, time pressure, and performance pressure.

Throw Outcome	Expertise	Condition			
		NOTP/NOPP	NOTP/PP	TP/NOPP	TP/PP
Hit	CE	−617.982 (76.96)	−724.137 (75.702)	−595.13 (81.888)	−581.808 (83.507)
	SE	−775.285 (105.4)	−695.28 (115.823)	−662.332 (117.533)	−668.324 (105.523)
Miss	CE	−785.191 (76.96)	−784.518 (78.513)	−682.497 (73.847)	−509.903 (73.088)
	SE	−746.578 (81.466)	−580.77 (79.863)	−588.525 (79.561)	−569.064 (81.492)

Note. A negative value represents a quiet eye onset before the critical movement (i.e., a greater value in absolute value correspond to an earlier quiet eye onset). Standard errors are presented inside the round brackets. Throw outcome (Hit; Miss); Expertise (CE = Competitive-elite; SE = Semi-elite); Time pressure (NOTP = No Time Pressure; TP = Time Pressure); Performance pressure (NOPP = No Performance Pressure; PP = Performance Pressure).

**Table A11:** Average quiet eye duration in milliseconds across throw outcome, expertise, time pressure, and performance pressure.

Throw Outcome	Expertise	Condition			
		NOTP/NOPP	NOTP/PP	TP/NOPP	TP/PP
Hit	CE	712.865 (82.791)	805.442 (81.574)	707.968 (87.593)	665.884 (89.174)
	SE	787.087 (111.765)	690.507 (122.059)	624.239 (123.74)	654.558 (111.888)
Miss	CE	851.576 (82.791)	860.578 (84.3)	762.975 (79.784)	637.662 (79.052)
	SE	801.38 (88.458)	584.907 (86.921)	567.075 (86.63)	566.588 (88.484)

Note. Standard errors are presented inside the round brackets. Throw outcome (Hit; Miss); Expertise (CE = Competitive-elite; SE = Semi-elite); Time pressure (NOTP = No Time Pressure; TP = Time Pressure); Performance pressure (NOPP = No Performance Pressure; PP = Performance Pressure).

**Table A12:** Average quiet eye offset in milliseconds across throw outcome, expertise, time pressure, and performance pressure.

Throw Outcome	Expertise	Condition			
		NOTP/NOPP	NOTP/PP	TP/NOPP	TP/PP
Hit	CE	95.27 (33.255)	81.494 (32.949)	112.338 (34.49)	86.152 (34.901)
	SE	14.178 (42.615)	−4.256 (45.403)	−39.25 (45.855)	−14.186 (42.65)
Miss	CE	65.998 (33.255)	75.845 (33.64)	80.822 (32.502)	126.467 (32.32)
	SE	53.837 (36.619)	3.989 (36.243)	−21.141 (36.171)	−2.305 (36.626)

Note. A negative value represents a quiet eye offset ended before the critical movement (i.e., a greater value in absolute value correspond to an earlier quiet eye offset). Standard errors are presented inside the round brackets. Throw outcome (Hit; Miss); Expertise (CE = Competitive-elite; SE = Semi-elite); Time pressure (NOTP = No Time Pressure; TP = Time Pressure); Performance pressure (NOPP = No Performance Pressure; PP = Performance Pressure).

**Table A13:** Average quiet eye preprogramming duration in milliseconds across throw outcome, expertise, time pressure, and performance pressure.

Throw Outcome	Expertise	Condition			
		NOTP/NOPP	NOTP/PP	TP/NOPP	TP/PP
Hit	CE	494.350 (82.013)	603.010 (79.108)	393.001 (84.676)	398.276 (90.629)
	SE	661.638 107.892)	553.010 (121.287)	497.613 (120.392)	563.390 (108.157)
Miss	CE	579.245 (77.711)	572.431 (79.452)	500.240 (74.365)	419.431 (80.224)
	SE	603.171 (84.690)	424.537 (83.138)	471.901 (83.258)	509.705 (87.754)

Note. Standard errors are presented inside the round brackets. Throw outcome (Hit; Miss); Expertise (CE = Competitive-elite; SE = Semi-elite); Time pressure (NOTP = No Time Pressure; TP = Time Pressure); Performance pressure (NOPP = No Performance Pressure; PP = Performance Pressure).

**Table A14:** Average quiet eye online duration in milliseconds across throw outcome, expertise, time pressure, and performance pressure.

Throw Outcome	Expertise	Condition			
		NOTP/NOPP	NOTP/PP	TP/NOPP	TP/PP
Hit	CE	386.740 (42.581)	388.454 (42.353)	378.659 (43.991)	464.759 (44.260)
	SE	285.777 (53.898)	305.124 (58.350)	242.300 (59.104)	228.001 (54.829)
Miss	CE	373.178 (42.581)	372.542 (42.971)	367.652 (41.945)	480.097 (41.712)
	SE	345.660 (47.614)	309.975 (47.280)	223.311 (47.207)	246.196 (47.686)

Note. Standard errors are presented inside the round brackets. Throw outcome (Hit; Miss); Expertise (CE = Competitive-elite; SE = Semi-elite); Time pressure (NOTP = No Time Pressure; TP = Time Pressure); Performance pressure (NOPP = No Performance Pressure; PP = Performance Pressure).
